# Opportunities and Challenges for Augmented Reality in Family Caregiving: Qualitative Video Elicitation Study

**DOI:** 10.2196/56916

**Published:** 2024-05-30

**Authors:** Liam Albright, Woojin Ko, Meyhaa Buvanesh, Harald Haraldsson, Fernanda Polubriaginof, Gilad J Kuperman, Michelle Levy, Madeline R Sterling, Nicola Dell, Deborah Estrin

**Affiliations:** 1 Department of Information Science Cornell University New York, NY United States; 2 Department of Computer Science Cornell Tech New York, NY United States; 3 Department of Information Science, Jacobs Technion-Cornell Institute Cornell Tech New York, NY United States; 4 XR Collaboratory Cornell Tech New York, NY United States; 5 Digital Informatics and Technology Solutions (DigITS) Memorial Sloan Kettering Cancer Center New York, NY United States; 6 Division of General Internal Medicine Department of Medicine Weill Cornell Medicine New York, NY United States

**Keywords:** augmented reality, extended reality, family caregiver, home care, virtual care, telemedicine, telehealth, oncology, artificial intelligence, mobile phone

## Abstract

**Background:**

Although family caregivers play a critical role in care delivery, research has shown that they face significant physical, emotional, and informational challenges. One promising avenue to address some of caregivers’ unmet needs is via the design of digital technologies that support caregivers’ complex portfolio of responsibilities. Augmented reality (AR) applications, specifically, offer new affordances to aid caregivers as they perform care tasks in the home.

**Objective:**

This study explored how AR might assist family caregivers with the delivery of home-based cancer care. The specific objectives were to shed light on challenges caregivers face where AR might help, investigate opportunities for AR to support caregivers, and understand the risks of AR exacerbating caregiver burdens.

**Methods:**

We conducted a qualitative video elicitation study with clinicians and caregivers. We created 3 video elicitations that offer ways in which AR might support caregivers as they perform often high-stakes, unfamiliar, and anxiety-inducing tasks in postsurgical cancer care: wound care, drain care, and rehabilitative exercise. The elicitations show functional AR applications built using Unity Technologies software and Microsoft Hololens2. Using elicitations enabled us to avoid rediscovering known usability issues with current AR technologies, allowing us to focus on high-level, substantive feedback on potential future roles for AR in caregiving. Moreover, it enabled nonintrusive exploration of the inherently sensitive in-home cancer care context.

**Results:**

We recruited 22 participants for our study: 15 clinicians (eg, oncologists and nurses) and 7 family caregivers. Our findings shed light on clinicians’ and caregivers’ perceptions of current information and communication challenges caregivers face as they perform important physical care tasks as part of cancer treatment plans. Most significant was the need to provide better and ongoing support for execution of caregiving tasks in situ, when and where the tasks need to be performed. Such support needs to be tailored to the specific needs of the patient, to the stress-impaired capacities of the caregiver, and to the time-constrained communication availability of clinicians. We uncover opportunities for AR technologies to potentially increase caregiver confidence and reduce anxiety by supporting the capture and review of images and videos and by improving communication with clinicians. However, our findings also suggest ways in which, if not deployed carefully, AR technologies might exacerbate caregivers’ already significant burdens.

**Conclusions:**

These findings can inform both the design of future AR devices, software, and applications and the design of caregiver support interventions based on already available technology and processes. Our study suggests that AR technologies and the affordances they provide (eg, tailored support, enhanced monitoring and task accuracy, and improved communications) should be considered as a part of an integrated care journey involving multiple stakeholders, changing information needs, and different communication channels that blend in-person and internet-based synchronous and asynchronous care, illness, and recovery.

## Introduction

### Background

Family caregivers are often recognized as the backbone of the health care system [1], providing vital care to millions of Americans [2]. Despite their importance in care delivery, research has shown that family caregivers face significant burdens [3,4] and do not receive sufficient support, recognition, or compensation [5-7]. Moreover, increased patient complexity, a push to decrease hospital lengths of stay, and shift toward outpatient care further burden family caregivers [3,8], including by increasingly requiring them to perform physical care tasks that clinicians (usually nurses) have traditionally performed, such as injections, tube feedings, catheter, colostomy, surgical drain care, and more [9,10].

One context where caregivers face substantial burdens is in cancer care. Many patients with cancer rely on family and friends to assist with cancer treatment needs, and family members often take primary responsibility as caregivers [11]. Being a caregiver for a patient with cancer requires multifaceted activities that are physically, emotionally, socially, and financially demanding [12,13] and has been shown to result in caregiver distress and poor quality of life [14,15]. Caregivers can feel overwhelmed [16], negatively impacting their own physical and mental health [15], which may in turn adversely affect patient health outcomes [17].

A promising avenue to reduce caregiver burden, including in cancer care, is via the design of digital technologies that provide caregivers with guidance and support [14]. For example, research has investigated mobile apps [18] and smart home sensing [19] to support caregiving, with efforts focusing on reducing caregiver stress [20], improving mental health [21], supporting remote caregiving [22], and more. There is also growing interest in the potential roles of extended reality technologies—an umbrella term for virtual, augmented, or mixed reality—to aid caregiving [23,24]. For example, research has proposed that using augmented reality (AR)—defined as a technology that superimposes a computer-generated image on a user’s view of the real world—could be useful for training caregivers [25,26] or connecting caregivers and remote clinicians [27,28].

### Objectives

Motivated by the challenges caregivers face delivering care at home and the opportunities for support offered by AR technologies, this study explores potential roles for AR to support caregivers. Specifically, we investigate how AR might support caregivers of patients with cancer as they perform physical tasks that are commonly required as patients with cancer recover from surgery and receive episodic treatments. In collaboration with cancer experts from Memorial Sloan Kettering Cancer Center (FP, GK, and ML), we identified examples of prevalent physical tasks common in cancer care: wound care, drain care, and physical rehabilitation. We chose to focus on physical care tasks because (1) they are increasingly required to be performed at home due to growing outpatient procedures and short hospital stays, (2) they require attention to dynamic patient-specific details, and (3) the hands-on nature of physical tasks makes this a promising area where the affordances of AR (eg, hands-free, overlaid graphical display) have the potential to be helpful.

We conducted a qualitative video elicitation study with caregivers and clinicians to explore how AR might aid the delivery of home-based cancer care. Our specific objectives were to (1) shed light on challenges caregivers face where AR might help, (2) investigate opportunities for AR to support caregivers, and (3) understand the risks of AR exacerbating caregiver burdens. Our findings could inform both the design of future AR devices, software, and applications and the design of caregiver support interventions based on already available technology and processes.

## Methods

### Video Elicitations for Cancer Care Tasks

#### Overview

To achieve our objectives, we conducted a qualitative video elicitation study with clinicians and caregivers. Video elicitation is a form of photo elicitation, a qualitative method where photographs, videos, or other visual media are used to elicit different kinds of knowledge than might be obtained via verbal interactions (eg, interviews) alone [29,30]. Video elicitation is especially valuable for exploring novel interaction techniques [31]. Relevant to our research context, video elicitation has proven effective for health care research [32].

Our study used video elicitations for several reasons. Creating videos enabled us to avoid rediscovering well-known issues with current AR technologies (eg, usability issues), allowing us to instead collect high-level, substantive feedback on potential future roles for AR in caregiving. Moreover, using video elicitations enabled exploration of a sensitive and fraught context—in-home caregiving for patients with cancer—without adding to participants’ current burdens or interfering with their caregiving or clinical responsibilities.

In partnership with cancer experts from Memorial Sloan Kettering Cancer Center, we identified physical caregiving challenges in the home. The specific tasks for the study were selected based on the experience of the clinical coauthors (FP, GK, and ML) who specialize in outpatient support and on publicly available materials (eg, pamphlets and websites) for home-based cancer care delivery. We created 3 video elicitations that offered examples of ways AR might support caregivers as they perform common—but often high-stakes, unfamiliar, and challenging—tasks in postsurgical oncology care: wound care, drain care, and rehabilitative exercise.

We note that these tasks are examples of common tasks that require advising by clinical staff, but they are not meant to represent a complete or prioritized list. Instead, they are intended to garner feedback on the general concept and opportunity, rather than being overly task specific or comprehensive.

The elicitations show functional AR applications built using Unity Technologies software and Microsoft Hololens2, which we selected for its optical see-through feature and well-supported software development environment. Optical see-through provides the most natural interaction and lowest latency. We intentionally designed the study to explore the potential utility of AR functionality, rather than focusing on the specifics of its implementation. The final elicitation videos range from 50 to 90 seconds in length and are provided in Multimedia Appendices 1-3. Next, we describe the videos in detail.

#### Wound Care

Wound care is a common task that caregivers perform for patients with cancer who have had surgery [33,34]. Wound care can be high risk; it is important to perform the task correctly to prevent infection [35] and to detect signs of infection as early as possible. Wound care can be anxiety inducing and stressful for caregivers [35,36] and may require communication with the care team to monitor and confirm the healing progress of the wound [37].

**Figure 1:**
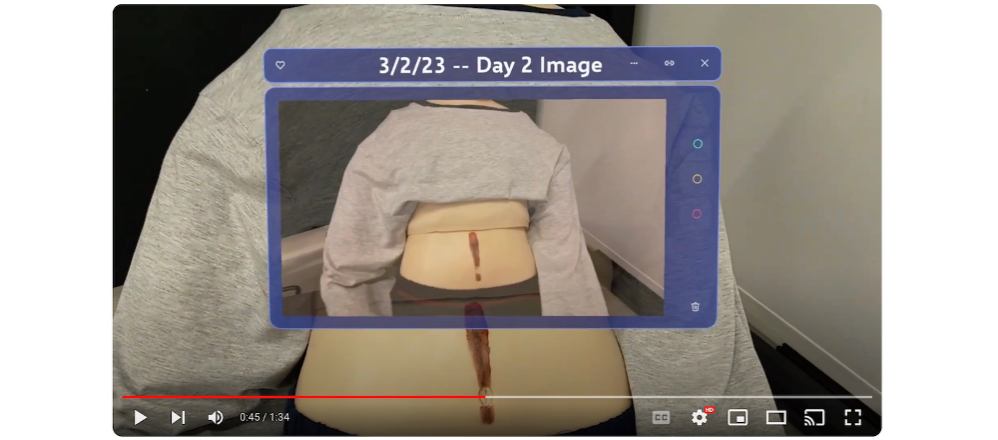
Caregiver’s view looking at the patient, with a captured photo of a wound shown in the augmented reality display.

We built a Microsoft HoloLens2 AR application that we then used in our elicitation (Figure 1). Key functionality we implemented to support the wound care task includes (1) drawing AR annotations on the field of view, (2) taking a photo of the wound using the HoloLens2 native camera, and (3) pulling up a photo gallery and comparing 2 photos side by side. The application is accessed from the HoloLens main menu by clicking on an icon. Once launched, the application is controlled by a visual menu bar rendered on the user’s wrist. The menu consists of a vertical list of 4 squares that contain icons corresponding to functions (eg, camera and drawing canvas).

**Figure 2:**
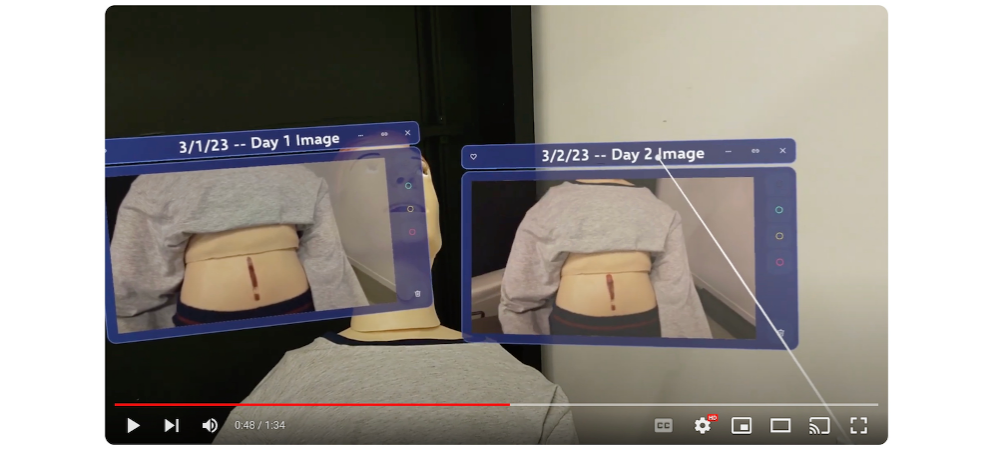
Caregiver’s view looking at the patient, with 2 photos of the wound taken on different days, enabling comparison.

After building the application, we recorded an elicitation video in which a researcher played the role of the caregiver, and a manikin was the patient. In the video, the caregiver (researcher) puts on the HoloLens2 headset and removes a bandage covering the patient’s (manikin’s) wound. Next, the video depicts what would happen on day 1, with the caregiver taking a picture of the wound (Figure 1). The video then progresses to day 2, where the caregiver pulls up the picture from day 1 and takes another picture of the wound on day 2. The caregiver puts the 2 images side by side and compares them visually (Figure 2). Perceiving a change in the condition of the wound, the caregiver opens the annotation feature and draws a line on the wound captured in the day 2 image, recording the length of the incision for further monitoring.

#### Drain Care

The second video elicitation focuses on drain care, another common task that can be unfamiliar and frightening for caregivers. A common type of apparatus used after breast cancer surgery, a Jackson-Pratt drain [38] consists of a plastic bulb and tubing that is connected to a suture area after surgery, with the goal of draining excess fluid and bodily material from the suture area [39]. For the Jackson-Pratt drain to function correctly, it must be routinely cleared to remove possible clogs and fluid buildup in the tubing. To do this, the caregiver or patient must perform a task called milking the tubing, which involves slowly pinching and pulling down the drain tube to push excess fluid or bodily material into the bulb. Failure to properly clear the tubing can lead to complications, including infection [40].

**Figure 3:**
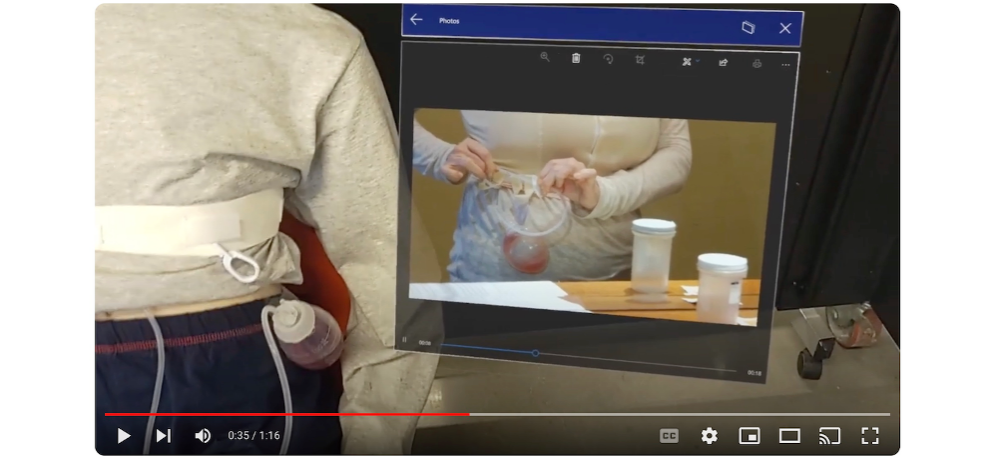
Caregiver’s view looking at the patient, with an instructional drain care video playing in the augmented reality display.

**Figure 4:**
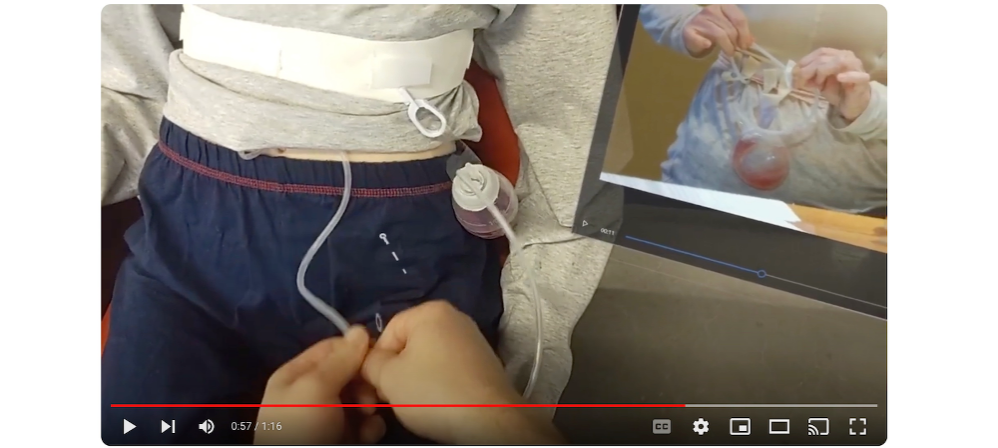
Caregiver’s view looking at the patient, while performing the drain care task after pausing the video in the augmented reality display.

The video elicitation for drain care focuses on enabling a caregiver to watch a Jackson-Pratt drain care instructional video on the HoloLens2 display, as they simultaneously perform the drain care task. The AR interactions involve moving a window by dragging and dropping, resizing the window, touching, and video playback. The elicitation starts with the caregiver putting the headset on and sitting next to the patient. It then switches to the caregiver’s point of view, with the Jackson-Pratt instructional video to the side of the field of view. The caregiver then drags and drops the drain care video so that it is next to the patient, resizes the video player window to be bigger, and starts playing the video (Figure 3). After a few seconds, the caregiver pauses the instructional video and begins the process of pushing, pulling, and clearing the drain (Figure 4). After several seconds of clearing the drain, the elicitation cuts to the patient’s view, showing the caregiver with the headset on clearing the drain. Finally, the elicitation switches back to the caregiver view, where they continue to go back-and-forth between the instructional video and the drain clearing task.

#### Physical Rehabilitation

The third elicitation focused on postsurgery rehabilitative exercises. After surgery, patients with breast cancer are often prescribed an exercise routine to maintain and build strength and functionality [41,42]. Failure to correctly perform these exercises can lead to a variety of negative health effects for the patient, including loss of mobility [43], muscle atrophy [44], ongoing discomfort [45], longer recovery times, and additional physician visits [46]. However, performing the required exercises can sometimes be uncomfortable and painful for patients, involving uncertainty about whether the exercises are being done correctly [47].

**Figure 5:**
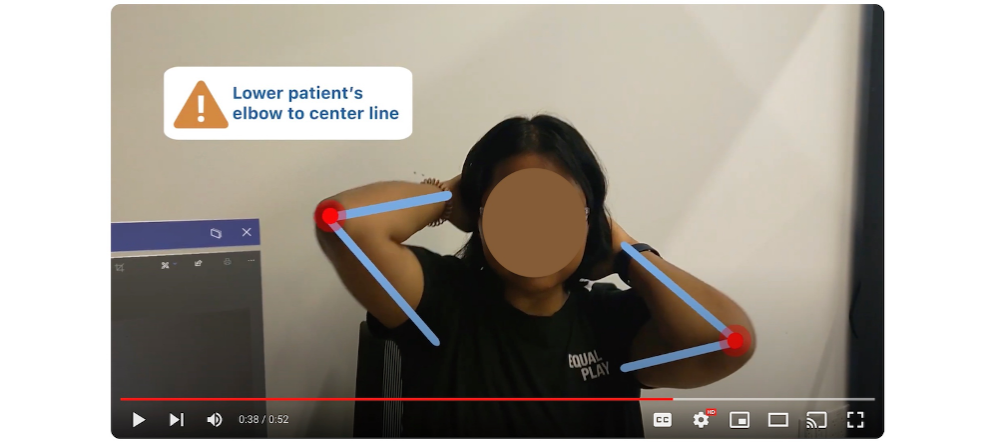
Caregiver’s view looking at the patient performing physical rehabilitation exercises, with the augmented reality tool display indicating incorrect movement.

To support rehabilitative exercises, we created an AR application that, by providing real-time feedback, enables caregivers to assess if a patient is correctly performing a required exercise (Figure 5). In addition to instructional video playback, the AR content includes graphical overlays (ie, lines) that track parts of the body (ie, arms). For simplicity, we achieved this effect via postproduction video editing (although others have implemented body tracking [48-50]).

**Figure 6:**
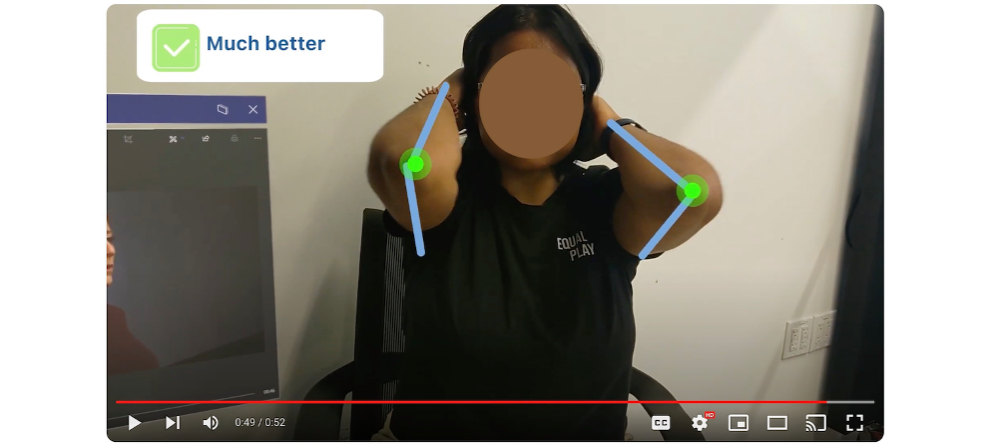
Caregiver’s view looking at the patient performing physical rehabilitation exercises, with the augmented reality tool indicating correct movement.

The elicitation starts with a shot of the caregiver’s hands pulling up an instructional exercise video next to the patient (played by a researcher in this scenario). The caregiver then starts the video, watching to see the correct exercise movement. The caregiver pauses the video and watches the patient perform the same exercise. The caregiver sees lines tracking the patient’s arms and dots signifying the patient’s joints. As the patient performs the exercise, their arms are not parallel (Figure 5); the caregiver sees the joint dots turn red and a warning sign pop-up saying, “lower the patient’s elbow to center line.” The caregiver adjusts the patient’s arms to the correct position, and the patient performs the exercise again. This time the dots on the joint turn green, and there is a pop-up in the field of view, notifying the caregiver that the exercise was performed correctly (Figure 6).

### Recruitment

After creating our elicitation videos, we recruited caregivers and clinicians for our study. Clinician participants were recruited from Memorial Sloan Kettering Cancer Center in New York City. Two researchers (FP and ML) sent individual emails that provided a synopsis of the study to oncology providers in the breast oncology service, with the goal of recruiting clinicians with diverse roles (surgeons, oncologists, nurses, etc). All participants currently work in cancer care delivery and have prior experience explaining oncology care plans to patients and caregivers. Clinicians who received a recruitment email and responded indicating interest in the study were then contacted by a member of the research team (FP and ML) to schedule an interview.

Caregiver participants were recruited via Cornell University email lists. One researcher (LA) sent email to the lists with a synopsis of the study that invited participation from anyone with experience working as a family caregiver. Interested participants who responded to the recruitment email were screened, with the inclusion criteria that they must have experience providing home-based care to a family member (ie, they did not need to possess experience in cancer care specifically). Participants meeting the inclusion criteria were scheduled for an interview at their convenience.

### Interview Procedure

One-on-one interviews were conducted in English via video conference software (Zoom Video Communications). Interviews lasted 60 minutes and were audio recorded. The interviewer (LA) introduced themselves, explained the study goals and interview format, and obtained informed consent. Then, participants were asked about their current and past involvement with caregiving. Questions sought an understanding of participants’ experiences, including information sharing and communication practices; challenges faced; and relevant training, resources, or support practices.

Next, the interviewer provided a brief overview of what AR is and asked the participant about their perceptions and any prior experiences with AR. Then, the interviewer shared their screen to show the elicitation videos. Participants watched the 3 elicitation videos one at a time. Before each video, the interviewer briefly stated what the scenario in the video showed (eg, “I’m now going to show you a video in which AR is used to support wound care”). After each elicitation video, the interviewer asked questions about the AR tool shown in the video and discussed participants’ perceptions of and reactions to it. The interviewer then moved on to the next video, with the order of presentation of the videos counterbalanced.

After the participant had watched and discussed all 3 videos, the interviewer asked for any concluding thoughts and asked the participants to complete an exit survey before ending the interview. All participants received a US $25 gift card as a token of appreciation for their time. The full interview protocol is provided in Multimedia Appendix 4.

### Data Analysis

Study data consisted of 22 hours of audio-recorded interview data that were professionally transcribed. Transcripts were uploaded into VERBI MAXQDA, a qualitative analysis software program. Transcripts were analyzed using a constructivist qualitative coding approach adapted from thematic coding [51], an established approach in human-computer interaction research [52]. This method involves developing descriptive codes via multiple passes over the data, with codes discussed and consolidated by the research team. Codes are then grouped into a set of themes that represent the data. In our analysis, interview transcripts were reviewed line by line and assigned codes. Multiple passes over the transcripts yielded a codebook of 40 unique codes (Multimedia Appendix 5), which were subsequently categorized into 11 themes (Textbox 1), with disagreements resolved through discussion.

**Summary of the study’s objectives and themes from the qualitative analysis.**

**Objectives and themes**

- Objective 1: shedding light on the challenges caregivers face where augmented reality (AR) might help
  Information overload
  Struggle to learn new care procedures and tasks
  Lack of tailored support
  Need to communicate with clinicians
- Objective 2: investigate how AR might support caregivers
  Enhance tracking and monitoring of a patient’s condition
  Improve task accuracy and adherence via real-time feedback
  Reduce communication barriers between clinicians and caregivers
  Reassure anxious caregivers
- Objective 3: understand how AR might exacerbate burdens
  Add to caregivers’ already heavy workloads
  Require training on how to use AR tools
  Introduce usability challenges

### Ethical Considerations

All participants provided informed consent to take part in the study and for their interview to be audio recorded. Cornell’s institutional review board determined the study to be exempt as part of protocol number IRB0008331.

## Results

### Participants

We recruited 22 participants: 7 caregivers (Table 1) and 15 clinicians (Table 2). Clinicians consisted of 8 women, 3 men, and 4 people who did not disclose their gender. All participants worked at Memorial Sloan Kettering Cancer Center. They possessed a range of specialties, including registered nurses, nurse practitioners, oncologists, and surgeons. All clinicians had prior clinical experience with oncology care, with an average of 15.7 (SD 9.1) years of clinical experience. The 7 caregiver participants consisted of 5 women, 1 man, and 1 person who did not disclose their gender. All caregivers had experience caring for a family member, ranging from a few weeks of postoperative care to >10 years of caring for a loved one with a chronic illness. Caregivers had an average of 4.6 (SD 5.6) years of experience providing home care.

**Table 1:** Prior experience of caregiver participants.

Number	Caregiving experience	Technologies used in caregiving
1	3 months	Desktop computer, paper, email, website, and mobile app
2	1 year	Laptop and paper
3	5 years	Smartphone, tablet, paper, phone calls, and mobile app
4	2 weeks	Smartphone and paper
5	7 years	Smartphone, tablet, desktop computer, paper, email, website, and mobile app
6	16 years	Smartphone, tablet, laptop, phone calls, email, and mobile app
7	>3 years	Smartphone, desktop computer, paper, and phone calls

**Table 2:** Occupational data and prior experience of clinician participants.

Number	Occupation	Years supporting patients with cancer	Technologies used to assist caregivers
1	Attending physician	31	Phone calls
2	Associate professor of surgery	11	Paper, videos, phone calls, email, website, mobile app, and video conferencing
3	Vice chair, department of medicine	20	Paper, phone calls, email, website, and video conferencing
4	Nurse practitioner	16	Paper and email
5	Registered nurse	32	Paper, phone calls, and website
6	Office practice nurse	9	Paper, videos, phone calls, email, website, and mobile app
7	Nurse practitioner	20	Paper, phone calls, and video conferencing
8	Registered nurse	9	Paper, videos, email, website, mobile app, and video conferencing
9	Advanced practice registered nurse	7	Paper, phone calls, email, website, and video conferencing
10	Registered nurse	8	Paper and email
11	Advanced practice registered nurse	7	Paper, email, website, mobile app, and video conferencing
12	Medical oncologist	30	Paper, phone calls, and video conferencing
13	Medical oncologist	7	Phone calls, website, and video conferencing
14	Registered nurse	17	Phone calls, website, and email
15	Medical oncologist	12	Paper, phone calls, website, and video conferencing

### Objective 1: Shedding Light on the Challenges Caregivers Face Where AR Might Help

#### Information Overload

When a patient receives their oncology care plan, it often includes chemotherapy treatment, an extensive drug regime, rehabilitation exercises, and contact resources. While trying to digest and parse all this information, caregivers are also processing the news that their loved one has a potentially fatal disease, a long treatment plan that could include invasive surgery, and complicated medication routines. All this means that caregivers struggle to digest information that they receive:

Pretty much as soon as you say the word chemo, people’s brains shut off...There’s a set spiel and I just acknowledge the fact that they’re not going to remember everything and that we’re just going to basically repeat it in several different ways, and hopefully, they can digest it.

Clinician 2

All the information related to the patient’s care plan is delivered via a collection of handouts and pamphlets given to the patient and caregiver, if present. This paperwork, which patients take home, can range from a few handouts to printed booklets, and the amount of paper can be significant:

I think what ends up happening is there’s too much information after a certain point. So, they take these handouts, and they stuff them in a folder which they have labeled their post-cancer treatment folder, and then they don’t look at them again.

Clinician 13

In addition to paper-based information, physicians and nurses also often provide spoken instructions or explanations to patients about their care plan and tasks that need to be performed. As caregivers may not always be able to attend clinical visits with patients, much of this information, whether spoken or paper pamphlets, may come secondhand to caregivers from patients. This may hamper caregivers’ ability to know what tasks need to be performed and how to perform them.

Our data also suggest that, for many caregivers, the challenges often become clear when the patient gets home. In addition to caring for the patient, caregivers often have other obligations, including career concerns or other dependents they need to care for, such as children. Juggling these responsibilities with their new care duties can be overwhelming:

When caregivers get home, that’s kind of where it all sinks in. Whether that’s realizing the volume of tasks that they have to do or how to even structure their day. Or it’s just really emotional for them.

Clinician 14

#### Struggle to Learn New Care Procedures and Tasks

Caregivers must learn the care duties they will need to perform. Sometimes, caregivers can attend in-person clinical visits with the patient. For care tasks that will need to be performed at home, the most crucial visits are often the preoperative and postoperative visits. In these visits, the patient and caregiver often sit with a clinician (eg, a nurse) and are provided pamphlets of information. Typically, clinicians physically demonstrate how to perform care tasks that will need to be done as part of the recovery plan. After the clinician demonstrates a task, the caregiver performs the task, with the clinician correcting any mistakes, and asking them to repeat the process (this is called the teach-back method):

I bring in a drain to show the [caregiver] before surgery what the drain looks like and what it feels like...so there’s some tactile stimulation there. We use the teach-back method here, so often I will show the patient or the caregiver how to empty the drain, and then I will say, “Can you show me how to empty the drain?” so I’m watching them teach me that, how to do it so that I can correct any behaviors that need to be changed and let them know the right way to do it.

Clinician 9

Whether using the teach-back method or spoken instruction, the ability to give real-time feedback and answer questions on the spot makes in-person learning the preferred setting for both caregivers and clinicians, as they can see body language and facial expressions, and the clinicians can quickly elucidate any missing information. In-person learning is especially useful for tasks that have a high degree of nuance or sensitivity. For instance, when clearing a drain, it might be difficult to know how much force to use without seeing an in-person demonstration that allows for real-time tactile feedback.

The overwhelming nature of the oncology process means caregivers can have trouble remembering everything they learned in the in-person visits. Moreover, in cases where the caregiver is unable to attend clinical sessions with the patient, they are often required to learn how to perform tasks at home from provided resources (ie, pamphlets) or from information provided secondhand by the patient. When the available resources prove insufficient, caregivers often search for information on the internet to help them:

A lot of people go to Dr. Google. They try to figure out what has worked for other people. So that can be a little frustrating, because we are trying to tell them, you know, this [booklet] is based on evidence.

Clinician 8

Sometimes the web-based information caregivers find is useful for learning how to do a care task, but information may also be inaccurate, and clinicians frequently do not know if caregivers have learned inaccurate information until the next check-in visit. Videos showing how to do tasks or providing examples of tasks being done can also be useful resources for caregivers:

A lot of people would prefer to see what they need to do. Sometimes, we have patients, when they need to do intricate dressing changes, sometimes we’re asked if they can video, they can record what we are doing.

Clinician 12

Caregivers often replay videos of the task being performed to help them learn and possibly correct mistakes based on information in the video that may not be apparent with paper instructions.

#### Lack of Tailored Support

We found that caregivers face increasing responsibilities but often encounter a lack of support tailored to their needs:

20 years ago, women that had a mastectomy would be in the hospital for seven days. Literally seven days. And now they’re out 23 hours later. So, family members have to do it now, as opposed to, you used to have physical therapy come in and teach them and do all of these things.

Clinician 11

Most existing support structures are targeted toward patients (ie, those receiving care), not caregivers. Thus, caregivers are often reliant on the information they get from the patient or spend substantial time and energy embedding themselves in the caregiving ecosystem (ie, going to physicians’ visits and logging into the patient portal using the patient’s credentials). The lack of tailored support for caregivers hampers their confidence, often leading to fear that they are performing tasks incorrectly and worry that any mistakes might hurt someone they care for:

My sister lives far away. She had surgery this year. My mother did the drains for the first few days. She’s a retired nurse. Worked fine. I was there to have a phone call, but my brother-in-law was freaking out. I had to walk him through everything on FaceTime and how to do it. He has no experience doing this. So, I just feel like it was almost like a virtual reality with me on FaceTime [helping] him do what he needed to do.

Clinician 11

The lack of tailored support systems for caregivers also means that care routines can be difficult to follow up on because, if tasks are being performed incorrectly, it may only be discovered during a follow-up clinic visit or when a patient’s condition worsens, and someone contacts the care team.

#### Need to Communicate With Clinicians

Our findings also suggest that caregivers currently struggle with disjointed communication systems. Caregivers often need to communicate with the clinical care team, including the patient’s physician, who creates and helps with the treatment plan, and nurses, who support the physicians and implement the care plan. Caregivers communicate with the care team in 2 main ways: through a web-based patient portal and via phone calls. Both these communication channels are again focused primarily on the patient, but caregivers often get informal access (eg, via the patient’s username and password). Communication with the care team happens for a variety of reasons. For example, if there is a problem with a medication or side effects, caregivers might send a message to the care team through the portal asking what to do. Alternatively, the patient’s condition might worsen, and the caregiver might call the care team to get help. Although participants reported that both the patient portal and phone calls were useful, they usually preferred phone calls:

I like picking up the phone and talking...it gives an opportunity for them to ask questions and answer back in real time. Where I think in the portal, things can get missed...Sometimes it’s a lot of back-and-forth.

Clinician 8

However, the web-based patient portal also had utility for participants. It can be challenging for caregivers to continually recall the essential information they need to perform tasks, and while web-based resources are easily searchable, they are often not verifiable. In contrast, the patient portal offers a viable way to provide information caregivers can refer back to:

I think the portal is good for relaying information that you want to become easily accessible for both the patient and the family, frankly, because it is a living record that allows them to kind of refer back to it.

Clinician 13

### Objective 2: Investigating How AR Might Support Caregivers

Having shed light on some of the challenges caregivers face where AR might help, we now discuss our participants’ reactions to the AR tools shown in our video elicitations. We start with how participants perceived that the interactive features in our elicitations might support their care tasks.

#### Enhance Tracking and Monitoring of a Patient’s Condition

One feature highlighted in our elicitations is the ability to view images and videos via the heads-up display, with content overlaid onto caregivers’ real-world view (Figure 1). A key benefit of overlaid content is that it enables caregivers to easily track changes in the patient’s condition by comparing what they are seeing with past data points:

Well, the overlay makes it much easier to do a comparison...Superimposing the images always gives you, at least to my mind, a better picture rather than when I have to go back and forth between two pictures. Going like this, trying to figure out if they’re different, is not the same thing as having everything in one field of view where you can do a direct comparison.

Clinician 2

Participants also liked how the annotation feature enabled directly drawing on the display, marking areas of interest for comparison or assessment. This was seen as especially valuable in the wound care scenario, where hand-drawn markings could be used to indicate areas of concern:

Oftentimes, if patients have an area of redness, as a nurse, we will just draw a line and see, is this beyond that? Where I think with this, you could take a photo, draw the line yourself, and then compare it the next day to see if redness, irritation, etc., extends beyond that line, helping make better assessments quicker rather than waiting for the patient to send you more information. I feel like from this standpoint, thinking of it just from a caregiver’s standpoint, I think that it can just provide a little bit better information in these types of assessments.

Clinician 15

Participants suggested adding features to the AR tool that might help caregivers to capture better photos. For example, in the wound care scenario, participants discussed how it can be challenging to get a clear and consistent picture, and they perceived that the AR tool could provide real-time feedback to ensure good lighting and consistent framing. Similarly, for the drain care scenario, participants imagined the tool could incorporate automated color correction and filtering capabilities:

Color really matters on both drain output and wounds. If the patient is sitting in direct sunlight or in the shade, understanding the image color is hard. If you can do some color filter to make it consistent, it would be easier for comparison.

Clinician 3

#### Improve Task Accuracy and Adherence via Real-Time Feedback

Participants said the AR overlays may also help caregivers to correct errors in real time as they perform tasks. With the rehabilitative exercise task in particular, participants noted the ability to have graphical overlays and annotations on the body as a key strength, communicating movement information clearly and effectively (Figure 6):

Specifically, because it gave them the angles, and it showed them how their body should look. And it also then kind of provided that feedback to them that they were doing it properly. And I think that that feedback loop for the patient and for the caregiver to see that, I think that’s super important because then the patient knows how it should look and how it should feel when they’re doing it properly.

Clinician 15

Participants also pointed out how graphical feedback may enhance accessibility for patients or caregivers who struggle to read or understand written instructions:

I also mentioned the language barrier. Right? So that might help folks like my parents, my mom doesn’t speak or read English. So, if she was able to see that, she would say okay, so I have to line up my arms. Great.

Caregiver 1

Participants also suggested enhancing accessibility by incorporating audio-based feedback into the AR tool. This would assist individuals who may struggle to process information visually, including older adults. Although the AR tools depicted in our video elicitations did not include any audio-based interaction, participants saw room for the tools to include this in the future. For example, participants imagined that the tool could audibly narrate step-by-step task instructions alongside the overlay and 3D annotation:

“Place your hands on it, and then in this manner, pull down.” Or however you want to say it...the audio would say, “Okay we will give you a few seconds to complete this part. Feel free to pause it if you need more time. Then we will go on to step two.” Yeah. I think it [would be] hard without the audio.

Clinician 10

#### Reduce Communication Barriers Between Clinicians and Caregivers

Participants also saw the potential for AR tools to help overcome some of their current communication challenges. Although our video elicitations did not explicitly include any communication between caregivers and clinicians, participants imagined, for example, being able to automatically upload captured data to a patient portal for monitoring and review by the clinical care team. Furthermore, participants pointed out that asynchronous monitoring of uploaded images may reduce the need for the patient to return to the clinic for a checkup if the care team can see their progress. This was seen as a major benefit as patients frequently live hours away from the clinic, and physically traveling to the clinic is a burden:

So, if you say this is what a typical wound looks like on day such and such. And this is what yours looks like. You can be like, oh, look at it. This looks okay. And you just continue to move through the continuum. And you’re sending the pictures anyway and someone is reviewing them. And as long as there’s not a problem, or you don’t need to reach out to the care team, then you would be okay. Then if it was an issue, then you might get a phone call to say we’ll look at them in real time and say what it is. If everything looks okay and there’s no concerns, then it would just be reviewed at the one visit, the postop visit or something.

Clinician 11

Another way clinicians might be able to see what a caregiver is seeing is if the AR tool enabled a shared point of view between caregivers and clinicians. Clinicians shared how, currently, it is challenging to understand the full context of what a caregiver is seeing. Sharing the caregiver’s point of view with the clinician via a camera embedded in the AR headset could enable clinicians to provide more effective feedback on task performance or teach caregivers to perform tasks. It would also enable clinicians to assess the patient’s condition remotely:

It lets you in the home. It lets you see—obviously, it’s not the same as examining the patient, but that’s probably the next best thing that you’re going to get. And you can also give education to the caregiver at the same time. So, it makes things just a little bit more efficient.

Clinician 14

However, while clinicians appreciated the potential utility of the tool, using the new technology may require providers to respond to patient and caregiver experiences using the new tool. This need to potentially help caregivers use the new tool may place additional burdens on clinicians.

#### Reassure Anxious Caregivers

Finally, participants saw the guidance and feedback provided by AR tools as a potential way to give valuable reassurance and validation to caregivers, who are often experiencing high levels of stress and anxiety as they try to care for a loved one. Receiving continuous encouragement and reassurance that they are performing tasks correctly may help to give them some peace of mind:

If there is a way for them to get reassurance that it’s going okay, then that would be useful. The great majority of people have never tried to deal with something bleeding sticking out of their wife’s chest. Most people haven’t dealt with that. It’s unpleasant. Something saying that you’re doing fine, it’s okay, this is going right, I think that reassurance would be helpful. I know it’s a low-level thing, but it’s very important.

Clinician 2

In contrast, other participants worried that AR tools might potentially increase anxiety for caregivers, especially those who are unfamiliar with AR and who may lack confidence in their ability to operate the technology correctly:

I wonder if, for someone who is not very familiar with [AR], if [the tools] might induce more anxiety, when they already have anxiety about doing a task for the first time. It may add one more complexity to an already somewhat complex situation.

Caregiver 3

We now discuss other ways in which introducing AR tools into caregiving ecosystems might unintentionally exacerbate caregivers’ burdens.

### Objective 3: Understanding How AR Might Exacerbate Burdens

#### Add to Caregivers’ Already Heavy Workloads

As discussed in detail in Objective 1, caregivers are often overwhelmed and face many challenges caring for a loved one. Introducing new technologies into complex caregiving contexts would require caregivers to balance learning and operating the new technology on top of the work required to perform the task at hand. Physically, caregivers would need to store and charge the device, boot it up, and start the relevant application. In addition, the novelty of AR interaction techniques and people’s general lack of familiarity using AR may result in a steep learning curve for caregivers:

They are not used to using this technology whatsoever. This would be new for them. I think they would feel like it’s almost like a little bit more of a hindrance because, you know, you’re talking about caring for somebody in the home and then they have to learn this whole new task of how to use the augmented reality. I think it might be a little overwhelming.

Clinician 10

Moreover, the high-stakes nature of caregiving contexts provides little room for error because any mistakes made by caregivers when performing tasks could lead to potentially negative health outcomes for patients. For example, errors when clearing a Jackson-Pratt drain can occur if pushing happens in the wrong direction or when too much pressure is applied to the drain, which may happen if the instructions provided by the AR tools are confusing or incorrect:

I could see that if the AR tool is giving someone instructions that aren’t correct, there’s that risk of injury...if the technology itself becomes a hindrance, that could interfere with the caregiving task.

Clinician 14

#### Require Training on How to Use AR Tools

To avoid potentially harmful mistakes, participants foresaw a need to equip caregivers with basic knowledge of how to use AR, along with training on our specific AR application’s interaction and use:

So, there’s many lessons needed within getting these tools to work: A, put this on. Next this is how you look around. This is how you use your hands with augmented reality...I guess when I think of this and scaffolding, I would think of both tools and modules to help the caregiver learn how to use augmented reality itself.

Clinician 14

The question of who would provide the relevant training for caregivers also arose, with clinicians concerned that it may be added to their already heavy workloads. Clinician participants discussed how they already balance phone calls, emails, and patient portal messages, and they were concerned that AR tools would further complicate their work assisting caregivers in the home:

You know the other thing that I’m thinking is that, just to be honest, like if I had to teach a patient how to use this, that’s going to be very time consuming. I honestly don’t think that I have time in my day to teach somebody. So, I’m not sure where this space is that that’s going to happen. For me to sit down and teach someone how to do this might take half an hour at least. I honestly don’t have that kind of time. So, I’m not sure where that would happen.

Clinician 10

#### Introduce Usability Challenges

Finally, participants imagined that the AR tools may be challenging to interact with, both in terms of general AR usability challenges and the usability of the specific AR applications we built. For example, it was not obvious to participants how they would discover and activate available features in the tool:

I was kind of like figuring out where does this go? And how does this?...I wasn’t quite sure where the camera button just automatically appeared from? How would that automatically appear? It wasn’t obvious to me where they went to get that.

Caregiver 3

Participants also worried that the annotation feature might be challenging for caregivers to use, particularly if the task required a precise drawing to correctly highlight a specific portion of the field of view. Participants also perceived that the field of view may quickly become too cluttered, adding to caregivers’ feelings of being overwhelmed:

It was too quick-moving. The size of the boxes could have been smaller. It was moving everything around so much.

Clinician 6

These findings highlight a need for careful, iterative design and usability testing with stakeholders before any attempts are made to integrate novel technologies such as AR into complex caregiving contexts.

## Discussion

### Principal Findings

Our qualitative study yielded rich insights into clinicians’ and caregivers’ perceptions of challenges caregivers face that might be ameliorated by AR. Most significant was the need to provide better and ongoing support for execution of caregiving tasks in situ, when and where the tasks need to be performed. Such support needs to be tailored to the physical needs of the patient, to the stress-impaired capacities of the caregiver, and to the time-constrained communication availability of clinicians.

The video elicitations that suggested how AR tools might support care tasks elicited valuable feedback and elaboration by study participants on two key affordances: (1) the ability to review prior image and video recordings as a way to enhance the caregiver’s ability to detect subtle changes in the patient’s condition and (2) the ability to share image and video recordings to receive timely feedback from clinicians on task execution. Participants suggested that these capabilities could contribute to improved communication between caregivers and clinicians, increase caregiver confidence, and lead to reduced anxiety. Opportunities for effective communication between caregivers and providers are increasingly important, particularly among adults with serious illness who often interact with multiple physicians, caregivers (family and paid), and health systems [53].

At the same time, the videos elicited reflection on potential exacerbations to caregiver burdens. For example, the introduction of new technologies into caregiving could increase the time and stress involved, requiring caregivers to carry out additional tasks and master the use of new tools, particularly given the significant usability limitations of the existing (demonstrated) AR headsets and the complexities associated with the use of these devices by untrained users in a home setting. These findings make clear that any attempt to integrate new technologies such as AR into caregiving contexts will require care and attention to potentially harmful unintended consequences. As discussed further, these insights are relevant and actionable in the context of recent developments in health care delivery and consumer technologies and could inform future research.

### Future Directions and Comparison to Prior Work

#### Extending Telemedicine

Over the past decade, the availability and capabilities of interactive, rich media communications—all the way from underlying wired and wireless bandwidth to end-user software and hardware platforms—made it possible for telemedicine (also referred to as virtual care) to be broadly usable and affordable. Then, urgent needs during the first year of the COVID-19 pandemic created a surge of adoption and reduction in regulatory barriers to telemedicine [54-56]. Now that we are past the crisis period, health systems, providers, and patients are exploring how, when, and where to use telemedicine in service of multistakeholder outcomes [57,58]. Despite its widespread adoption, research acknowledges limitations in today’s most prevalent telemedicine systems, which rely primarily on synchronous, interactive video sessions and chat capabilities [59]. Some of this prior research also surfaced evidence that caregivers play a significant role in telemedicine encounters and follow-up [27,60,61].

The AR capabilities we investigated have the potential to extend telemedicine systems [62]. A critical feature of these capabilities is that they do not depend on continuous or synchronous interaction between caregivers and clinicians. Instead, caregivers can use the technology to support their work performing physical care tasks when and where they need it, with input available at the time of their work, not at the mercy of the availability of the clinician. At the same time, the technology can enable asynchronous communication and reporting to clinicians via transmission of photos or videos that, if necessary, can be reviewed by clinicians at their convenience. This may enable clinicians to be more effective by allowing them to fit caregiver feedback and questions into their busy schedules. At the same time, such asynchronous interactions could contribute additional burden to clinicians and provider organizations more generally.

It is important to note that our study presents early explorations into potential roles for AR in caregiving. Before AR tools can be deployed or adopted in practice, future work is needed to explore how these tools might be validated to ensure safety and quality of care in practice. This would require both validation or assurance of the software itself (ie, that the tools will not dangerously malfunction due to software bugs) and validation that assures quality of care and accurate interpretation of information provided to caregivers. Such validation will be key to ensure safety and minimize risks to patients and caregivers.

Moreover, our study suggests that the AR technologies and the affordances they provide should not be viewed as isolated tools. Rather, they should be considered as a part of an integrated care journey involving multiple stakeholders, changing information needs, and different communication channels that naturally blend in-person and virtual synchronous and asynchronous care, illness, and recovery.

#### Media-Rich Consumer Devices

At a high level, our findings suggest that the current generation of AR headsets is not yet ready for use by untrained caregivers in a home setting. While AR technologies continue to be developed, we see ample opportunities for existing consumer technologies to support, at least partially, some of the affordances our participants valued.

Most obvious, perhaps, is the opportunity to do image and video capture, review, sharing, and annotation by using standard mobile devices (eg, smartphones and tablet devices). Many mobile-based software programs provide the ability to capture and annotate images and videos. Moreover, aspects of AR overlay can be developed using AR capabilities currently available on mobile devices. However, unlike a head-mounted display, the use of mobile devices will only be applicable for tasks and contexts where hands-free use is not required because the user needs to hold the mobile device in their hands. Nonetheless, a variety of tasks may benefit from this mode of interaction.

#### Investment and Innovation in Caregiving

More generally, and importantly, this work joins calls for increased investment and innovation focused on caregivers [6,63,64]. As our study and prior research [65-67] show, caregivers’ emotional, informational, and communication needs are frequently not explicitly considered and are instead ignored or conflated with patients’ needs. However, caregivers, for example, may have different perspectives than patients and may be better able to monitor patient progress than patients themselves. Explicitly integrating caregivers into the information processes and hierarchies of clinical care could lead to benefits and improved outcomes for all stakeholders [68]. This research is also aligned with new federal policies that aim to reduce caregiver burdens, including the Recognize, Assist, Include, Support, and Engage Family Caregivers Act [69] and the National Strategy to Support Family Caregivers [70]. Our study also joins calls for improved caregiver training, upskilling, and compensation [71,72] by highlighting the opportunity to treat caregivers as key agents for incorporation of new tools, technologies, and methods to improve home-based care.

### Limitations

As a qualitative study, this research is subject to several limitations inherent to qualitative research [73,74]. For example, the study involved a relatively small sample of participants recruited in a single academic institution and high-volume cancer center in an urban area. Participation was also limited to clinicians and caregivers, and we did not collect clinical information about the patients for whom the caregiver participants provided care, including type of cancer, severity of disease, and functional and cognitive status. As such, the findings may not generalize to other regions, contexts, or stakeholders (generalizability is typically not a goal of qualitative research [74]). Additional research is needed to understand the perspectives of different populations, including in rural settings. Further research is also needed to investigate the important perspectives of patients who are care recipients, especially patients who are receiving cancer care.

The video elicitation methods we used have additional limitations. For example, although the videos depicted real applications built using a commercial AR device (Microsoft HoloLens2), participants did not directly put on and use the headset or interact with the AR applications themselves. Thus, there may be further challenges associated with usability of AR technologies for performing care tasks that were not surfaced by our study. Similarly, given that our interviews used a 2D video, we do not claim that our findings cover both the benefits and challenges specific to 3D interactions. Such issues would need to be explored in future work, with future versions of 3D AR devices, as meaningful experiments will be highly dependent on the specifics of the device’s user experience.

### Conclusions

We conducted a qualitative video elicitation study with caregivers and clinicians to explore the potential for AR technologies to aid caregivers’ delivery of home-based cancer care. Our findings shed light on clinicians’ and caregivers’ perceptions of the current information and communication challenges caregivers face as they perform physical care tasks as part of cancer treatment plans. We uncover opportunities for AR technologies to potentially increase caregiver confidence and reduce anxiety by supporting the capture and review of images and videos and by improving communication with clinicians. However, our findings also suggest ways in which, if not deployed carefully, AR technologies might exacerbate caregivers’ already significant burdens. On the basis of these findings, we discuss future directions for the research and design of technologies such as AR to aid the delivery of care at home.

## Appendix

Multimedia Appendix 1 Elicitation video showing how augmented reality might support wound care.

Multimedia Appendix 2 Elicitation video showing how augmented reality might support drain care.

Multimedia Appendix 3 Elicitation video showing how augmented reality might support rehabilitative exercise.

Multimedia Appendix 4 Interview protocol, showing questions asked to clinician participants in black. If the questions were modified for caregiver participants, the modified question wording is provided in blue.

Multimedia Appendix 5 Codebook from the qualitative video elicitation study showing, for each objective, the corresponding themes and codes.

## Abbreviations

AR: augmented reality
