# Supplementary material for: Opportunities and Challenges for Augmented Reality in Family Caregiving: Qualitative Video Elicitation Study
Source: JMIR Form Res. 2024 May 30;8:e56916. doi: 10.2196/56916 (PMC11176885; doi:10.2196/56916)
Supplement: Multimedia Appendix 4 [file formative_v8i1e56916_app4.docx]

Table: Interview protocol, showing questions asked to clinician participants are in black. If the questions were modified for caregiver participants, the modified question wording is provided in blue.

| **Topic** | **Clinician Questions/**  **Caregiver Questions (if different)** | **Clinician Probes /**  **Caregiver Probes (if different)** |
| --- | --- | --- |
| How do caregivers get information on caregiving tasks performed in the home | What post-surgical information resources have you found caregivers use?    What post-surgical information resources do you use? | Do you give caregivers/patients formal material from the clinical team?  Do you as a caregiver receive formal material from the clinical team? |
|  |  | Do caregivers bring in their own information resources to the caregiving routine in the home? Is this common/rare?  Do you bring in your own information resources to the caregiving routine in the home? |
|  |  | Do caregiver support groups (e.g. a Facebook cancer support group) share information relating to specific care tasks? Can you describe how you've seen resources shared?  Are you a part of any caregiver support groups if so do you share/gather information there? |
|  |  | How does this information differ from the formal clinical guides? |
|  | How common is it for caregivers to encounter issues when performing care tasks using the information provided?  How does this information differ from the formal clinical guides? | After reviewing the caregiving instructions from a hospital discharge clinician, what follow-up questions do caregivers ask?  After reviewing the caregiving instructions from a hospital discharge clinician, what follow-up questions do you ask? |
|  |  | How often do caregivers seek assistance with medical care tasks?  How often do you seek assistance with medical care tasks? |
|  |  | Which events, if any, trigger caregivers to seek help with tasks?  Which events, if any, trigger you to seek help with care tasks? |
|  | Which types of caregivers (children/siblings/friends/other) seek more information and/or encounter more issues along the way? |  |
|  | What practices best support caregivers' retention and use of information?  What practices best support your retention and use of caregiving information? |  |
|  | Do you have analytics/data on caregiver information website usage? |  |
| What types of information do caregivers receive | Do different types of caregivers prefer specific formats(paper/pen/email/text ..etc) of communication or information sharing?  What specific formats (paper/pen/email/text ..etc) of communication or information sharing do you prefer? | Are there specific caregiving videos you recommend for people?  Are specific formats more effective for certain tasks than others? |
|  |  | How are physical therapy instructions given to patients/caregivers?  What format do you prefer physical therapy instructions in? |
|  |  | How would you characterize patient preference with different formats (paper? graphical? text messaging? photo share...)? |
|  |  | Are specific formats more effective for certain tasks than others? |
|  |  | Is there a caregiver task-tracking system? |
|  | How do caregivers use the Recovery Tracker? | How common is it for caregivers to fill the tracker out for or with patients? |
|  |  | Do patients or caregivers access the recovery tracker from their phones? |
|  |  | Do you routinely use a recovery tracker? |
| Information/  Instructional complications | What happens when caregiving tasks (e.g. Wounds / Ports / Drains /Mobility/medication) have not been performed correctly? How is this detected?  What happens when caregiving tasks (e.g. Wounds / Ports / Drains /Mobility) have not been performed correctly? How is this detected? |  |
|  | What are some of the reasons that caregivers struggle with following instructional material? (literacy, stress, overwhelmed...)  What are some of the reasons that people struggle with following instructional material? (literacy, stress, overwhelmed...) |  |
|  | Have you observed caregiver's modifications to clinical home care instructions? If so, how? |  |
| Introduction to AR | How familiar would you say you are with Augmented Reality Technology? |  |
|  | Have you used an augmented reality application before? | Have you heard of PokemonGO, IKEA Place, or SnapChat AR? |
|  |  | How much have you used it? |
|  |  | What do you use it for? |
|  |  |  |
| AR tool questions | What are your initial thoughts about the Augmented Reality (AR) tool demonstrated in the videos? | How might the AR tool affect a caregiver's ability to do their job? |
|  |  |  |
|  |  | For which types of caregiving tasks would the tool be useful or not useful for? |
|  |  |  |
|  |  | What additional capabilities would you be interested in? |
|  |  |  |
|  |  | What concerns do you have about the AR tool in the video? |
|  |  |  |
|  |  | How would you feel about a caregiver using this device? |
|  |  |  |
|  | What parts of using such an AR tool do you anticipate being problematic (e.g., tedious, annoying, difficult, inefficient?) |  |
|  |  |  |
|  | What other kinds of tasks (beyond rehab or wound care) might benefit from an AR tool? |  |
|  |  |  |
|  | What challenges do you foresee coming up if these tools were put into practice? |  |
|  |  |  |
|  | Do you have any final thoughts on the tool demonstrated? |  |
|  |  |  |
