# Supplementary material for: Opportunities and Challenges for Augmented Reality in Family Caregiving: Qualitative Video Elicitation Study
Source: JMIR Form Res. 2024 May 30;8:e56916. doi: 10.2196/56916 (PMC11176885; doi:10.2196/56916)
Supplement: Multimedia Appendix 5 [file formative_v8i1e56916_app5.docx]

**Multimedia Appendix 5.** Codebook from the qualitative video elicitation study showing, for each objective, the corresponding themes and codes.

| Theme | Codes |
| --- | --- |
|  |  |
| **Objective 1: Shedding light on caregivers’ current realities** | |
| Feeling overwhelmed | Current Issues: overwhelm  Current Issues: information overload  Information Materials: handout  Current Issues: Challenges with information retention |
| Struggle to learn new care procedures and tasks | Video Instruction: replay  Information Sharing: in person education  Clinician Preference: teach hospital  Information Search Behavior |
| Lack tailored support | Current Issues: Lack of support in home  Care Task: Incorrect image  Care Tas: Follow Up  Current Issues: Not following care routine  Current Issues: Care task fear |
| Need to communicate with clinicians | Patient Communication: Phone call  Patient Communication: Portal  Current Issues: Patient portal |
| **Objective 2: How AR might support caregivers** | |
| Enhance tracking and monitoring of a patient’s condition | AR Capability: Overlay  AR Capability: Better Images  AR Usefulness: 3D Annotations  AR Usefulness: Shared POV  AR Usefulness: Future applications |
| Improve task accuracy and adherence via real-time feedback | AR Capability: Realtime feedback  AR Capability: Audio  AR Capability: Multiple users |
| Reduce communication barriers between clinicians and caregivers | AR Usefulness: Reduce doctor visits  AR Usefulness: improve communication  AR Usefulness: More documentation |
| Reassure anxious caregivers | AR Usefulness: reduce anxiety  AR Usefulness: General |
| **Objective 3: How AR might exacerbate burdens** | |
| Add to caregivers’ already heavy workloads | AR Negative Outcomes: Increase anxiety  AR Negative Outcomes: Inaccurate assistance  AR Negative Outcomes: Increase work burden  AR Negative Outcomes: motion sickness |
| Require training on how to use AR tools | AR Appropriateness: Complex procedures  AR Compared to phone  AR Feasibility: Increased training  XR familiarity |
| Introduce usability challenges | AR Feasibility: Clean clear interface  AR Feasibility: Logistics  AR Feasibility: Interactions difficult |
